# Calcium-phosphate ceramics and polysaccharide-based hydrogel scaffolds combined with mesenchymal stem cell differently support bone repair in rats

**DOI:** 10.1007/s10856-016-5839-6

**Published:** 2017-01-21

**Authors:** Sophie Frasca, Françoise Norol, Catherine Le Visage, Jean-Marc Collombet, Didier Letourneur, Xavier Holy, Elhadi Sari Ali

**Affiliations:** 1 Département Soutien Médico-Chirurgical des Forces, Institut de Recherche Biomédicale des Armées (IRBA), BP 73, 91223 Brétigny-sur-Orge cedex, France; 2 AP-HP, Service de Biothérapie, Hôpital de la Pitié Salpêtrière, Paris, France; 3 INSERM U791, Centre for Osteoarticular and Dental Tissue Engineering, Nantes, France; 4 INSERM U1148, LVTS, Université Paris 13, Hôpital X. Bichat, Université Paris Diderot, Paris, France; 5 AP-HP, Département de Chirurgie Orthopédique et Traumatologie, Hôpital de la Pitié Salpêtrière, Paris, France

## Abstract

Research in bone tissue engineering is focused on the development of alternatives to autologous bone grafts for bone reconstruction. Although multiple stem cell-based products and biomaterials are currently being investigated, comparative studies are rarely achieved to evaluate the most appropriate approach in this context. Here, we aimed to compare different clinically relevant bone tissue engineering methods and evaluated the kinetic repair and the bone healing efficiency supported by mesenchymal stem cells and two different biomaterials, a new hydrogel scaffold and a commercial hydroxyapatite/tricalcium phosphate ceramic, alone or in combination.

Syngeneic mesenchymal stem cells (5 × 10^5^) and macroporous biphasic calcium phosphate ceramic granules (Calciresorb C35^®^, Ceraver) or porous pullulan/dextran-based hydrogel scaffold were implanted alone or combined in a drilled-hole bone defect in rats. Using quantitative microtomography measurements and qualitative histological examinations, their osteogenic properties were evaluated 7, 30, and 90 days after implantation. Three months after surgery, only minimal repair was evidenced in control rats while newly mineralized bone was massively observed in animals treated with either hydrogels (bone volume/tissue volume = 20%) or ceramics (bone volume/tissue volume = 26%). Repair mechanism and resorption kinetics were strikingly different: rapidly-resorbed hydrogels induced a dense bone mineralization from the edges of the defect while ceramics triggered newly woven bone formation in close contact with the ceramic surface that remained unresorbed. Delivery of mesenchymal stem cells in combination with these biomaterials enhanced both bone healing (>20%) and neovascularization after 1 month, mainly in hydrogel.

Osteogenic and angiogenic properties combined with rapid resorption make hydrogels a promising alternative to ceramics for bone repair by cell therapy.

## Introduction

Bone reconstruction after tumors, traumas or pathologies is a common challenge encountered in regenerative medicine. To date, autologous bone graft is the gold standard to treat such injuries but this method is greatly restricted by important morbidities related to the bone graft collection procedure [1] and there is a crucial need for developing new bone substitutes. In recent years, a better understanding of the biological process underlying bone tissue repair led to approaches based on a combination of scaffolds with osteoprogenitor cells.

Scaffolds must be selected for their ability to optimize bone healing, promote cell survival, proliferation and differentiation and must be nonimmunogeneic, while exhibiting appropriate degradation, mechanical strength and flexibility properties. Most commonly approved biomaterials are hydroxyapatite (HA) and tricalcium phosphate (TCP)-mixed scaffolds according to their natural bone mineral similarities and their biocompatibility and bioreactivity. However, HA/TCP ceramics exhibit extensive in situ resorption latencies preventing the gradual replacement with newly formed bone [2]. Biomaterial design is expanding with new material syntheses, including synthetic polymers, fibrous scaffold, bioactive ceramics, metals, composite scaffolds, and processing techniques to enhance the complexity of 3D environments [3–5]. A growing interest for polymer hydrogels to enhance bone healing is arising on the basis of their easy shaping capacity, radio-transparency and high resorption ability.

Multiple stem cell-based products have been used in humans for tissue regeneration. Mesenchymal stem cells (MSCs) are promising candidates and this is particularly true within the field of bone regeneration since they differentiate into osteoblasts, the mature cells responsible for bone formation. Their great potential in regenerative medicine also lies on their in vitro expansion ability as well as their anti-inflammatory and pro-angiogenic properties. If the physiology and the differentiation ability of MSCs have been extensively studied in vitro, the fate of these progenitors during in vivo bone metabolism and bone repair processes remains poorly understood [6, 7].

Several investigations suggested that natural bone healing response involves the mobilization of endogenous MSCs from bone marrow to the site of injury and their subsequent differentiation into osteoblasts to participate in the bone repair process. This natural bone healing mechanism can be potentially enhanced by administering exogenous cultured MSCs combined with artificial scaffolds to bone defect [8–10]. Thus, Granero–Molto et al. showed in a stabilized tibia fracture mouse model that transplanted MSCs migrate to the fracture site, contribute to the repair process initiation and have a key role in the inflammatory response, thus participating to each fracture healing stages [11]. Li et al. confirmed this contribution of transplanted MSCs in a mouse model of osteogenesis imperfecta [12]. They speculated that transplanted cells induced differentiation or recruitment of endogenous cells to initiate reparative process at early stages of bone repair. Several animal studies have evidenced the MSC and biomaterials-osteogenic properties and some clinical studies have suggested a beneficial effect of HA/TCP ceramics colonized with MSCs on bone repair in patients [9, 13–16]. Despite these valuable progresses, bone tissue engineering is not part of routine clinical practice, underlying the need for further animal and clinical investigations to define optimal combinations biomaterial/osteoprogenitor cells and understand their mechanisms of action in the bone healing process.

The present study compared the bone healing process induced with a porous pullulan/dextran-based hydrogel scaffold that has already successfully been used in vitro for cardiovascular engineering applications [17–19] or a commercial HA/TCP ceramic, alone or combined with MSCs, in a rat femoral drilled-hole bone defect. Microtomography and histology analysis were used to compare their respective efficiency up to 3 months after implantation.

## Materials and methods

### Culture of rat bone marrow MSCs

Bone marrow was flushed through the medullary cavity of femurs collected from syngeneic Lewis rats. Collected bone marrow cells were expanded in minimal alpha medium (αMEM; Gibco) supplemented with 1% penicillin/streptomycin (Life Technologies, France), 10% fetal bovine serum (Hyclone; Thermoscientific), and 1 ng/mL basic-fibroblast growth factor (bFGF; Peprotech, France) in an incubator at 37 °C with 5% CO_2_ and 95% humidity. Plastic-adherent cells (*i.e.* MSCs) were subcultured every 4–7 days, and then characterized by flow cytometry analysis using phycoerythrin-labeled anti-CD45 (Immunotech) and fluorescein isothiocyanate (FITC)-labeled anti-CD90 (Becton Dickinson) antibodies. MSCs were also characterized by their capacity to differentiate along adipogenic, chondrogenic, and osteoblastic lineages as previously specified [20].

Quantum dot^®^-labeled MSCs were transplanted to our experimental rat models to perform in vivo cell tracking study. Quantum dot^®^ nanocrystals integrate the MSCs cytoplasm and exhibit intense photostable fluorescence in vivo for at least 4 months [21].

### Preparation of implants

**Fig. 1:**
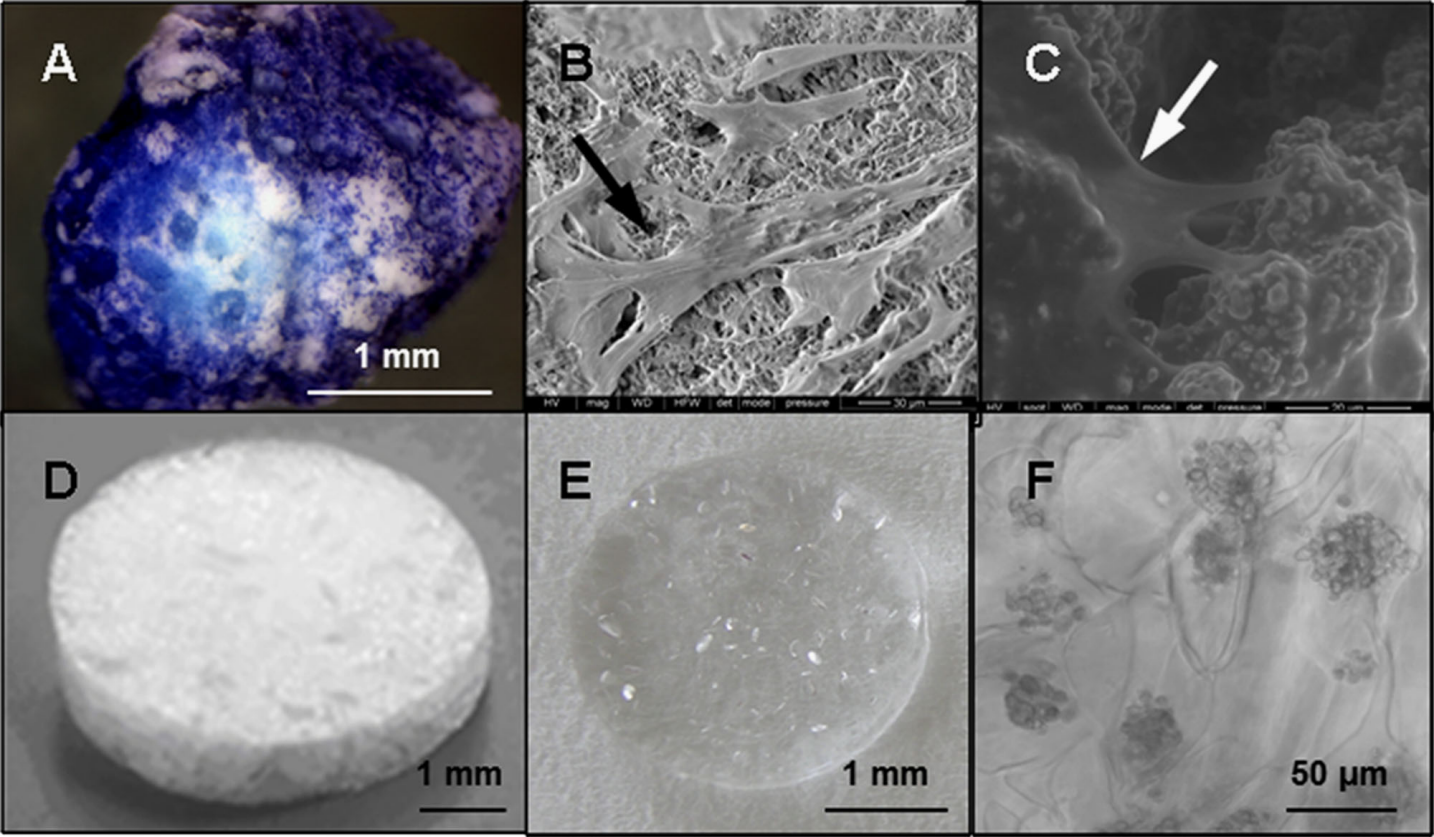
Assessment of MSC-scaffolds colonization. Scaffolds were seeded with 5.10^5^ rat MSCs. After 4 days of culture, the MSCs colonization of Calcirecorb35^®^ granule is confirmed by Trypan blue staining (**a)**, or scanning electron microscopy at the granule surface (**b)** and at macropore entrance (**c)**. Dehydrated porous polysaccharide scaffold (**d)** was seeded with MSCs immediately before implantation and cell infiltration within the transparent hydrogel (**e)** was assessed microscopically after 10 min, with cell clusters observed within the hydrogel pores (**f)**. *Arrows* indicate cells on/in biomaterials

Macroporous biphasic calcium phosphate ceramic granules (Calciresorb C35^®^, HA/TCP = 65/35) were obtained from Ceraver, France (Fig. 1a–c). To promote cell adhesion on granules, 5 × 10^5^ harvested MSCs were suspended in 200 µL αMEM culture medium and transferred into a tube containing a single C35 granule. After 2 h in a 37 °C incubator, granules with adherent MSCs were placed into 6-well plates and cultured for 4 days prior implantation.

Polysaccharide-based hydrogel scaffolds were synthesized and characterized as previously described [22]. Briefly, hydrogels were prepared using a mix of pullulan (MW 200,000; Hayashibara) and dextran (MW 500,000; Sigma) in distilled water. Chemical cross-linking of these polysaccharides was carried out using the cross-linking reagent sodium trimetaphosphate (STMP; Sigma) under alkaline conditions, with addition of porogen reagent sodium carbonate (Na_2_CO_3_, Sigma). Pore size and interconnectivity were selected in order to optimize cell infiltration [17]. We demonstrated that calcium carbonate porogen agent caused the formation of large pores of about 200 μm, favorable for MSCs infiltration [22, 23] while sodium chloride would create smaller pores (40 μm) that would allow seeding of smaller cells such as endothelial cells [24]. On this basis, we produced 200 μm diameter pores, round-shaped porous scaffolds of 6 mm diameter and 1 mm thickness (Fig. 1d), cellularized with 5 × 10^5^ MSCs in 20 μL αMEM culture medium (15 min, 37 °C) immediately before surgical implantation.

### In vivo implantations

All animal treatment and procedures were approved by the Institutional Animal Care and Research Advisory Committee of IRBA in accordance with French law and main international guidelines. Adult male Lewis rats (Janvier, Le Genest-St-Isle; France) weighing 220–250 g were bilaterally implanted for 7, 30, and 90 days, providing 10 samples per biomaterial condition and experimental time.

- “Control” group with no specific treatment;
- “MSC” group with 5 × 10^5^ rat MSCs in 20 µL culture medium;
- “Hydrogel” group with culture medium-hydrated hydrogel;
- “Hydrogel + MSC” group with hydrogel cellularized with 5 × 10^5^ rat MSCs;
- “C35” group with culture medium-hydrated calciresorb35^®^ granules;
- “C35 + MSC” group with calciresorb35^®^ cellularized with 5 × 10^5^ rat MSCs.

Defects were achieved by drilling a 3 mm diameter hole through the anterolateral cortical bone into the metaphyseal cancellous bone marrow, under continuous irrigation with saline. Osseous cavities were carefully filled with the different implants and then, muscles and skin were sutured in different layers (Vicryl^®^4/0). Analgesia was achieved through subcutaneous injections of buprenorphine hydrochloride (30 µg/kg, Buprecare, Animalcare, UK) 2 h after surgery and twice a day over three consecutive days.

All rats were sacrificed by overdose injections of sodium pentobarbital (Dolethal, Vétoquinol, France), then femurs were collected and fixed in 4% paraformaldehyde for X-ray microtomography (µCT) and histological analysis.

To measure mineral apposition rate (MAR) at day 30, calcein fluorochrom (75 mg/kg, Merck) was intraperitoneally injected to rats, 12 and 3 days before sacrifice. Calcein is incorporated in the mineralization front by the time of injection [25].

#### X-ray microtomography (µCT) analysis

Femurs were scanned using a SkyScan 1174 tomograph (SkyScan, Belgium) with the following parameter setup: source energy at 50 keV, intensity of 800 µA and isotropic voxel resolution of 15 µm with a 0.5 mm depth aluminum filter. After 3D reconstructions with Nrecon V1.4 software (SkyScan, Belgium), bone structure was analyzed using CTan software (SkyScan, Belgium). The newly mineralized bone volume fraction in the defect cavity was defined as the BV/TV parameter (Bone Volume/Tissue Volume ratio). For C35 ceramics, global segmentation was determined in order to separate newly mineralized elements from C35 ceramics background using the CTan software histogram tool to threshold gray level values.

#### Histological examinations

Following µCT scanning, undecalcified paraformaldehyde-fixed femurs were successively dehydrated in graded ethanol solutions and xylene. Then, femurs were embedded in Technovit^®^ resin (Heraeus Kulzer GmbH, Wehrheim, Germany) for 5 days at −20 °C. Serial 5 µm-thick longitudinal sections were obtained (Leica microtome, Denmark) and stained with Masson–Goldner’s trichrome to identify bone structures, fibrous tissue and bone marrow cells. Alcian blue dye allowed hydrogel fragments identification. Staining for bone specific-alkaline phosphatase (ALP) and tartrate-resistant acidic phosphatase (TRAP) activities were performed to reveal mature osteoblasts and osteoclasts, respectively [26, 27]. Stained sections were imaged on a DMRB microscope (Leica) connected to a Sony DXC930 color video camera. To analyze ALP and TRAP activities and blood vessel density, 5 consecutive sections were randomly chosen. From each section, neovascularization and ALP positive osteoblasts or TRAP positive osteoclasts were estimated in the randomly chosen field of 500 µm^2^ on a semiquantitative scale: (0) None; (1) Low; (2) High by 2 blinded pathologists. For some samples at day 30, number of vessels was manually counted in the defect area.

Detection of Quantum Dot^®^-labeled MSCs and MAR measurements on bone sections were achieved using a fluorescence microscope (Olympus IX71, Melville, NY) connected to a spot Sony SE digital camera. For MAR measurements (µm/day), the distance between the two fluorescent calcein lines (corresponding to the position of the mineralization front by the time of the calcein injections) was measured using a semi-automatic image analyzer software (Histolab, Microvision, France). As a control, MAR was determined at a distance >3 mm from the defect site.

#### Statistical analysis

For each experimental group, values are expressed as mean ± standard error of the mean (SEM). Statistical comparisons were made by using one or two ways analysis of variance (ANOVA) tests for MAR and BV/TV values, respectively. Statistical differences were considered as significant when *P* values < 0.05. Considered parameters for BV/TV statistical analysis are the experimental time and the bone defect treatment. Whenever ANOVA yielded significant interaction difference, a Tukey’s HSD post-hoc test was thus performed. A statistical software package R 3.0.1 (Vienna, Austria) was used to achieve statistical comparisons in this study.

## Results

### In vitro colonization of scaffolds by MSCs

After a gentle apposition and 4 days of rat MSCs culture, C35 granules were massively colonized by cells as assessed by trypan blue staining (Fig. 1a) and scanning electron microscopy (Fig. 1b, c). MSCs were preferentially localized on the C35 surface or near the pore entrances.

Clear and transparent hydrated hydrogels (Fig. 1e) allowed for a direct observation of large MSCs clusters spotted inside the hydrogel pores (50–200 µm diameters, Fig. 1f), in the entire thickness of the scaffold thus validating the instantaneous cellularization of hydrogels with MSCs.

### 3D micro-computed tomography analysis

**Fig. 2:**
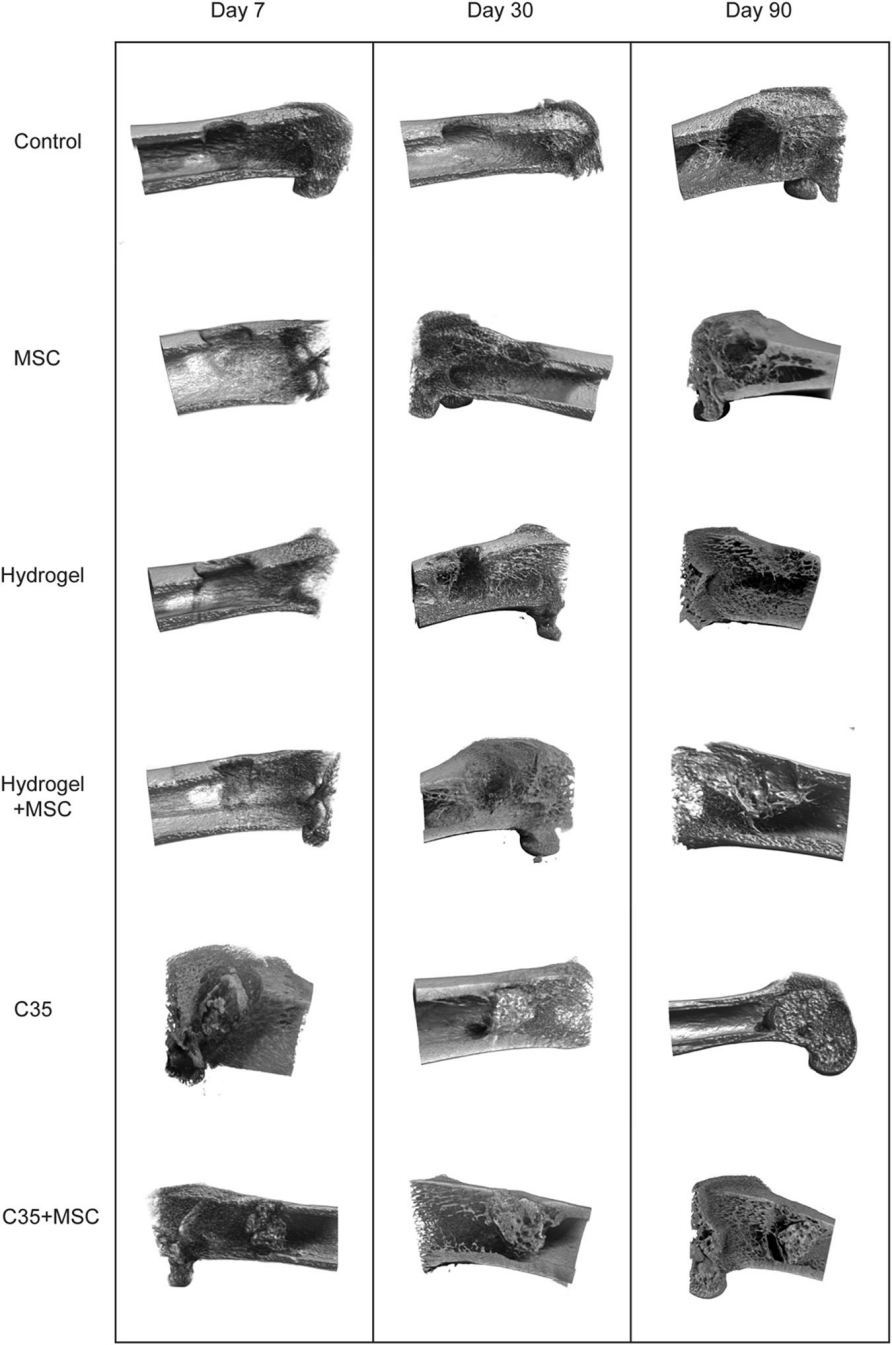
Representative 3D micro-CT images of the rat femoral distal end for each group on days 7, 30, and 90. Untreated defects showed very few mineralization within the defect even after 3 months. Hydrogels induced a cortical bone-like mineralization on the sides of the defect as early as post-surgery day 7. C35 were partially covered with new bone on post-surgery day 30

Figure 2 shows representative µCT scan images of the bone defect cavity illustrating bone healing progression on post-surgery day 7, 30, and 90 in all experimental groups. The control group generated negligible mineralized tissue within the defect cavity, up to day 90. The absence of any cortical bone restoration was also clearly evidenced. MSCs administration enhanced bone formation and was characterized by the development of bony spikes as early as 30 days after implantation. Furthermore, a partial closure of the cortical defect was achieved with MSCs on day 90.

A different bone repair pattern was noticed depending on the nature of the implanted scaffold. Hydrogels combined or not with MSCs induced a cortical bone-like mineralization on the edges of the defect as early as day 7 and this bone formation pathway was sustained up to day 90. In addition, cancellous bone-like components were detected in the cavity center. The C35 ceramics associated or not with MSCs supported newly mineralized bone around granule surfaces on day 30. On day 90, some internal pores of the granules appeared to be partially filled with newly synthesized bone but C35 ceramics failed to be resorbed.

**Fig. 3:**
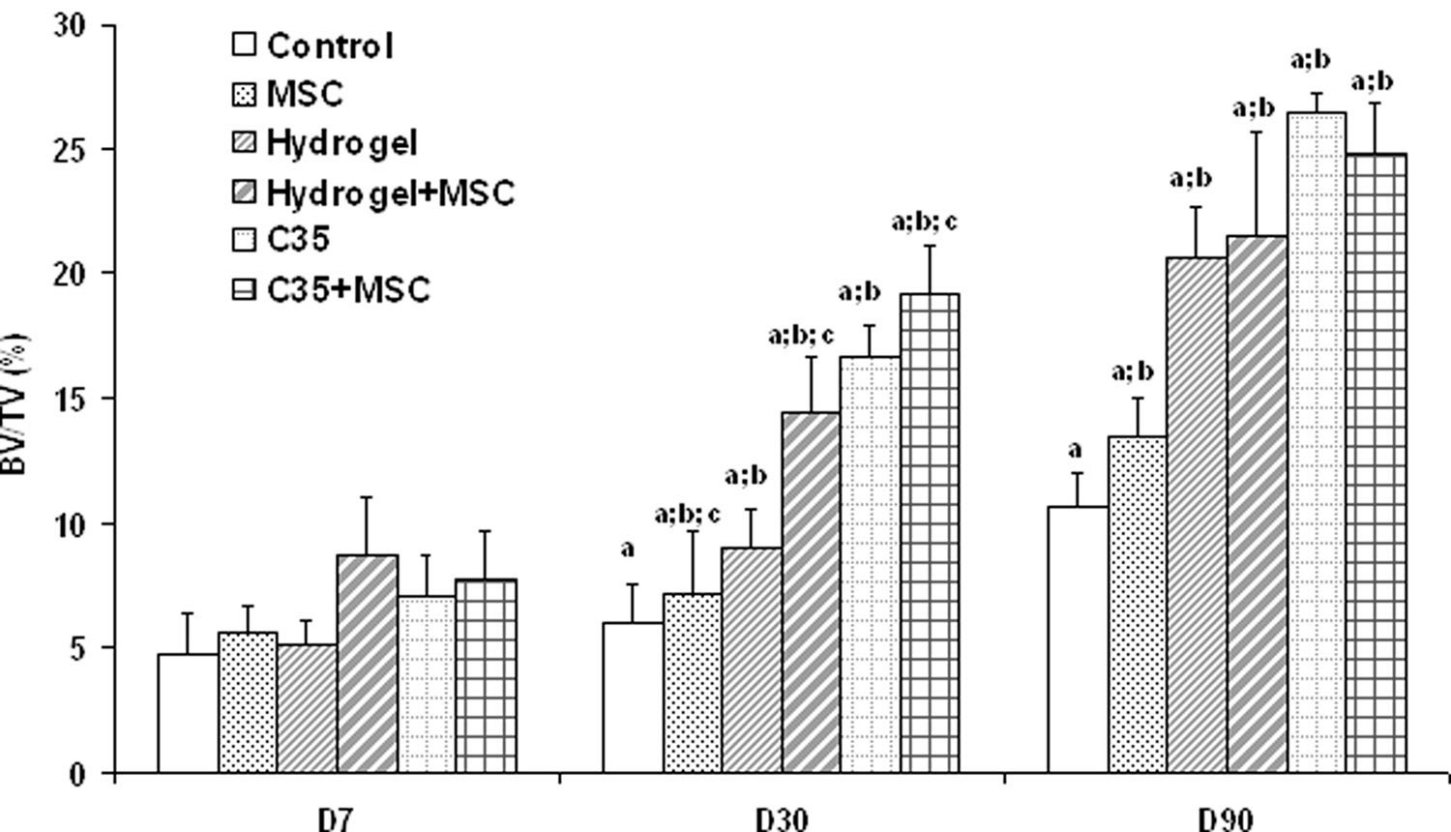
Bone volume fraction (BV/TV) for each group at 7, 30, and 90 days after surgery in the defect area. Given values are the mean BV/TV ± SEM for each experimental group. Significant differences (*p* < 0.05) when comparing **a**: effect of time within a considered group. **b**: each group to its respective control group for a defined experimental time. **c**: each group with MSCs to its respective group without MSCs for a defined experimental time

The µCT scan allowed quantifying the newly synthesized bone in the medullary cavity (Fig. 3). On day 7, BV/TV values in the medullary cavities were similar in all experimental groups (from 4 to 9%). On day 30, BV/TV for the control group remained unchanged (6.0 ± 2.5%) when compared to day 7 (4.7 ± 2.7%). Interestingly, the implantation of both scaffolds significantly increased medullary cavity BV/TV values, reaching 16.6 ± 1.7% with C35 ceramics (*p* = 0.004) and 9.0 ± 2.6% with hydrogels (*p* = 0.049) on day 30. At this time, MSCs delivery induced a significant increase in BV/TV values (*p* = 0.017 with ANOVA two parameters) by +20% in control group (“control” vs. “MSC”), +16% in C35 group (“C35” vs. “C35 + MSC”) and +61% in hydrogel group (“Hydrogel” vs. “Hydrogel + MSC”).

On day 90, the BV/TV value in the medullary cavity of control rats was only 10.6 ± 1.9% highlighting the inefficiency of the natural bone healing process to restore the original bone integrity. To the opposite, both tested scaffolds exhibited impressive osteoconductive properties since the augmentation of BV/TV values was sustained, reaching 26.5 ± 0.5% for C35 ceramics (*p *< 0.001) and 20.6 ± 3.9% for hydrogel (*p *< 0.001). The initial addition of MSCs failed to significantly modify the BV/TV values in the medullary cavity of control and biomaterial-treated rat femurs at this time.

### Histological studies

**Fig. 4:**
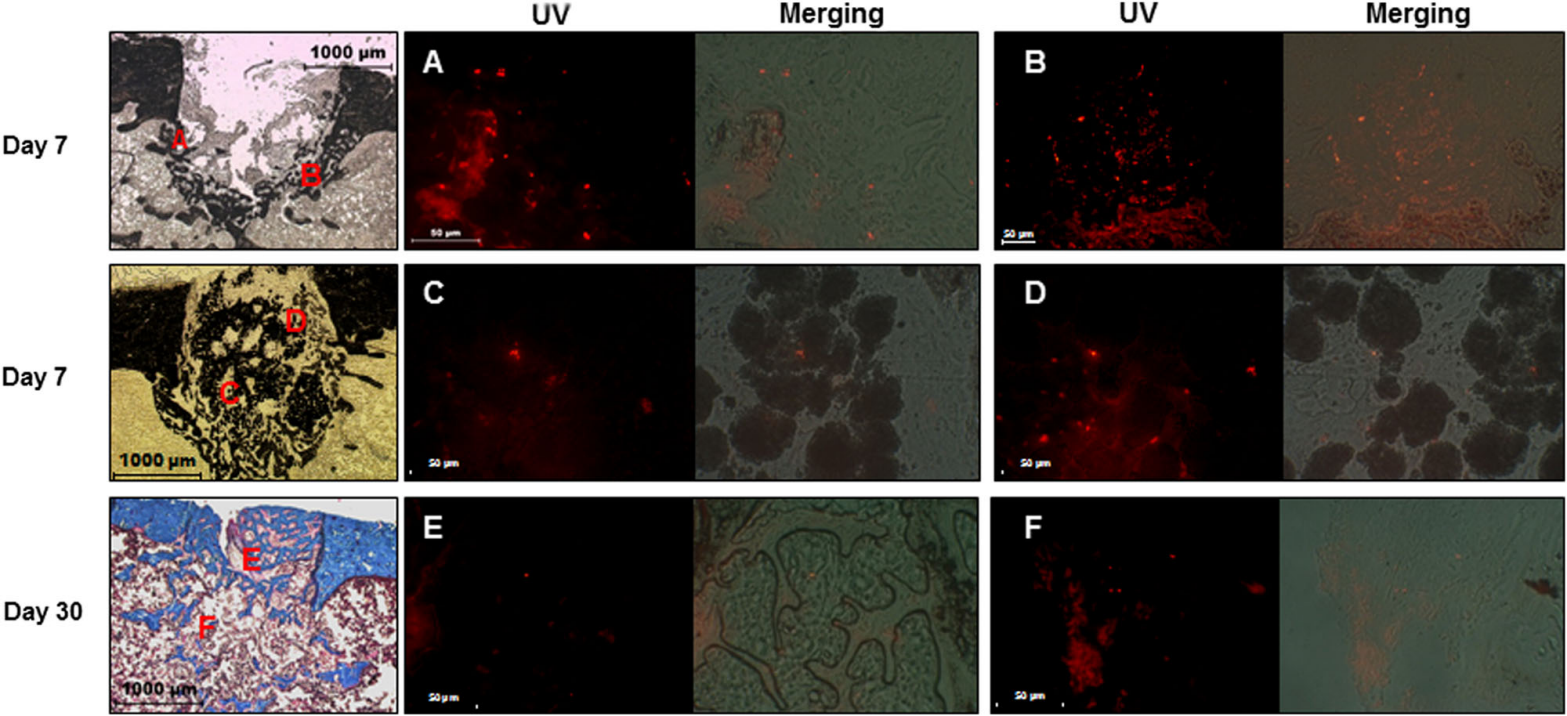
Fluorescent staining of MSCs in the defect area 7 days (Von Kossa staining, bone in *black*) and 30 days (Masson-Goldner’s trichrome staining, bone in *blue*) after surgery. Cells were labeled with quantum dots prior to implantation. Merging of photomicrographs obtained under normal light or under UV excitation with a specific filter allows the detection of labeled cells close to the edges of the defect both in control and hydrogel groups at day 7 (**a**, **b**), or close to ceramic surfaces at day 7 (**c**, **d**), and both in new bone (**e**) and fibrous tissue (**f**) at day 30

Quantum dot^®^-labeling gave important clues on the distribution of delivered MSCs within the bone defect. Both in untreated defect and hydrogel group, labeled MSCs were observed in the bone defect on day 7 mainly located on the edges of the defect (Fig. 4a, b), or close to the ceramic surfaces (Fig. 4c, d). On day 30, engrafted MSCs were sparser within the entire defect area. Some labeled cell clusters appeared to be entrapped in the bone matrix of the newly synthesized bone components (Fig. 4e, f). On day 90, labeled MSCs were not detected anymore in the bone defect area.

**Fig. 5:**
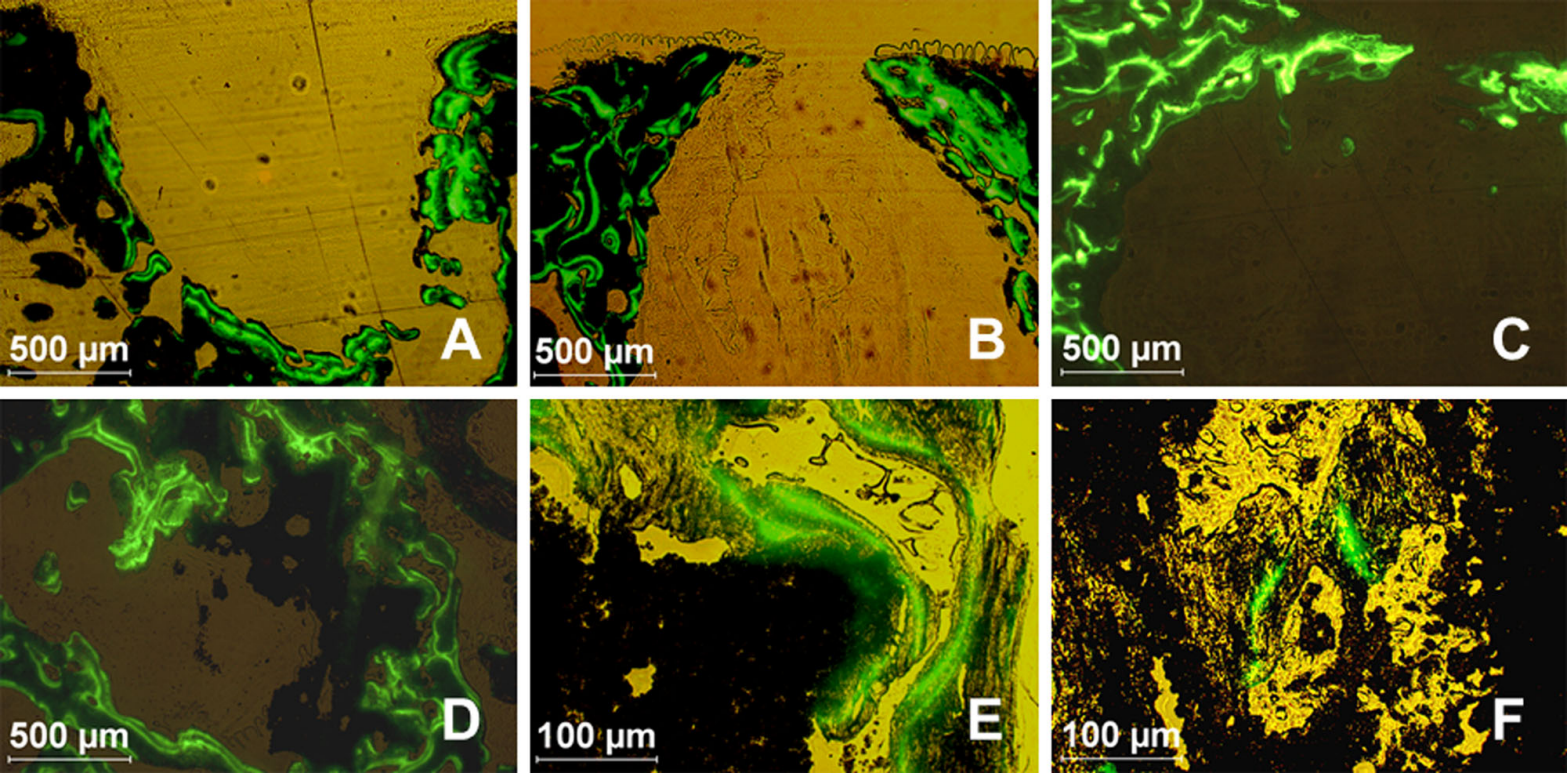
Calcein labeling of mineralization fronts in the defect area for each group with MSCs 30 days after surgery**.** Injections of fluorochrome were performed 12 and 3 days before sacrifice. **a** Control; **b** MSC; **c** Hydrogel + MSC; **d** C35 + MSC; Magnification of (*D*) on the C35 surface **e** and in a pore **f**

The presence of ALP positive osteoblasts was investigated in all six groups. Some were detected on the cavity sides as well as on newly synthesized bone trabeculae in the defect area whatever the considered experimental times, but only in the MSC-containing groups (semi-quantitative scoring = 1). Furthermore, MAR values which reflect the rate of new bone deposition, and thus indicate the speed of repair, were similar, ranging between 3.8 and 5.2 µm/day, independently of the considered experimental groups, in medullary cavities and in unlesioned bony areas of all rats (Fig. 5).

**Fig. 6:**
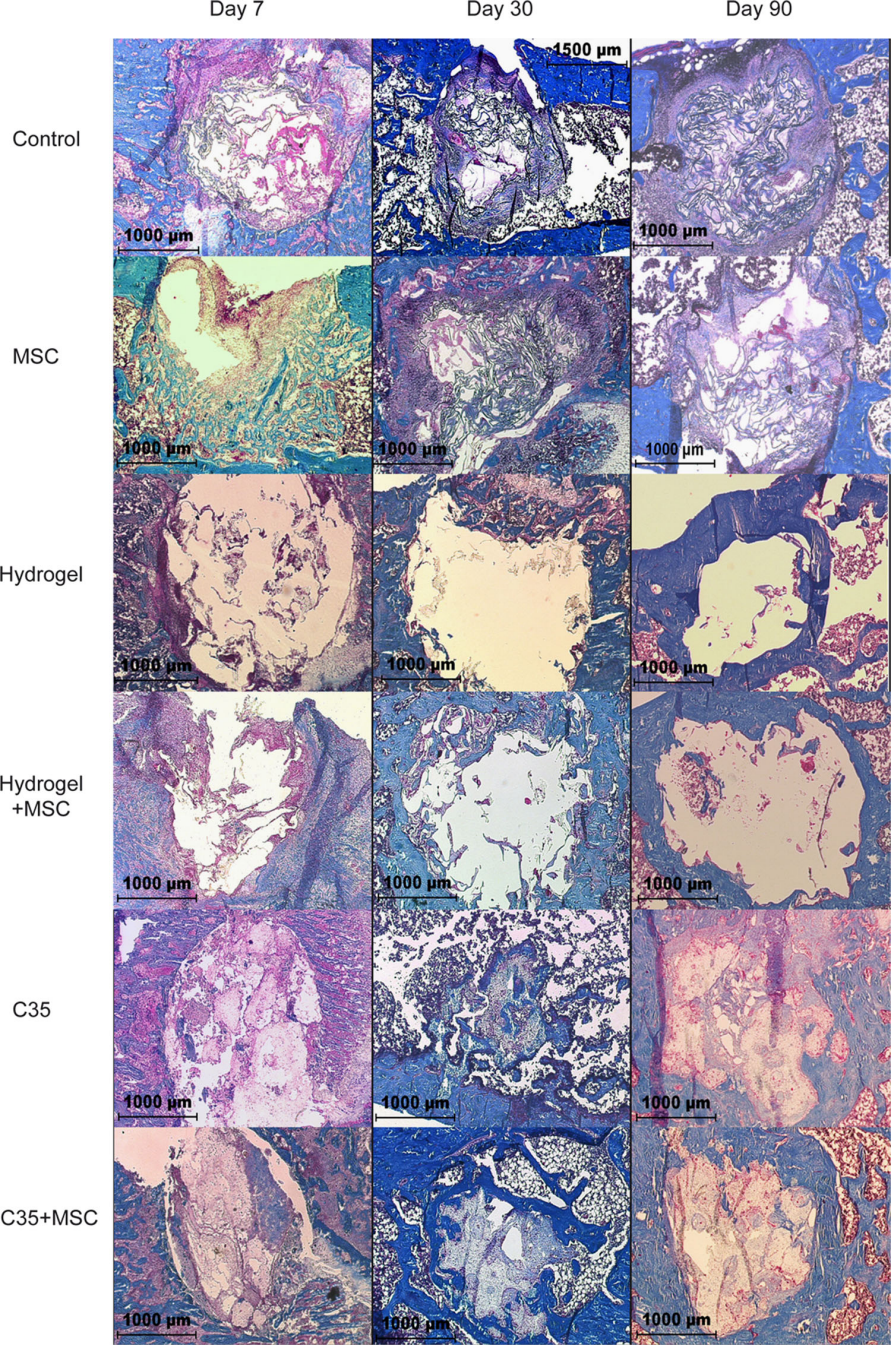
Representative histological sections of Masson–Goldner’s trichrome-stained undecalcified rat femoral defects implanted with hydrogels or C35 ceramics with or without MSCs on days 7, 30, and 90. Mineralized tissue is *blue*, fibrous tissue is *red*/*pink*, ceramics have a *white shadowy* appearance. Note that depending on the cutting angle, one cannot see the defect opening

Masson–Goldner’s trichrome staining confirm data observed by µCT scan image analysis concerning newly mineralized bone and provide additive information on the nature of non-mineralized tissue in the medullary cavity (Fig. 6). As an overall comment, histological analyses excluded the presence of any cartilaginous tissue formation or endochondral ossification, thus suggesting an exclusive intramembranous bone formation pattern in all animal groups. From day 0 to day 90, a minimal bone healing with a prominence of poorly vascularized fibrous connective tissue in the medullary cavity of control rats was observed. MSC group supported bone repair as characterized by a partial closing of the cortical defect and the presence of newly synthesized trabeculae, however restricted to the edges of the defect.

With ceramics, newly-mineralized deposits were spotted on granule surfaces on day 30 and the thickness of mineralized tissue increased on day 90. In medullary cavity areas not occupied by ceramic granules, typical bone marrow cells were shown in association with rare little trabecular-like spikes. Interestingly, newly formed bone in the C35 macropores was exclusively detected when MSCs were combined to C35 granules. Mineral deposits around granule surfaces were associated to double calcein layers while in internal pores a unique calcein layer was observed suggesting a delayed mineralization (Fig. 5e, f).

When using the hydrogel as a bone repair support, a large amount of fibrous tissue was found surrounding the gel in the medullary cavity on day 7. On day 30, large bone filling with newly regenerated bone marrow cells was achieved. At this time, an important bone mineralization occurred at the medullary cavity periphery leading to the formation of a thick shell-like compact bone structure (Figs 6 and 8). Newly-synthesized trabecular bony spikes were also detected within the cavity area.

According to the semiquantitative scale evaluation, neovascularization was not detectable in control animals and those administered with MSCs, hydrogel or ceramic alone. When hydrogel was combined to MSCs, neovascularization was detected at day 30, at scale 2 using semi-quantitative method and 2.4 ± 1.6 vessels/mm^2^ in samples submitted to manual counting; there was a trend to increase at day 90 (3.7 ± 2.6 vessels/mm^2^), while at this time, vessel number was estimated to scale 1 (0.5 ± 0.2 vessels/mm^2^) after implantation of MSCs with ceramics.

**Fig. 7:**
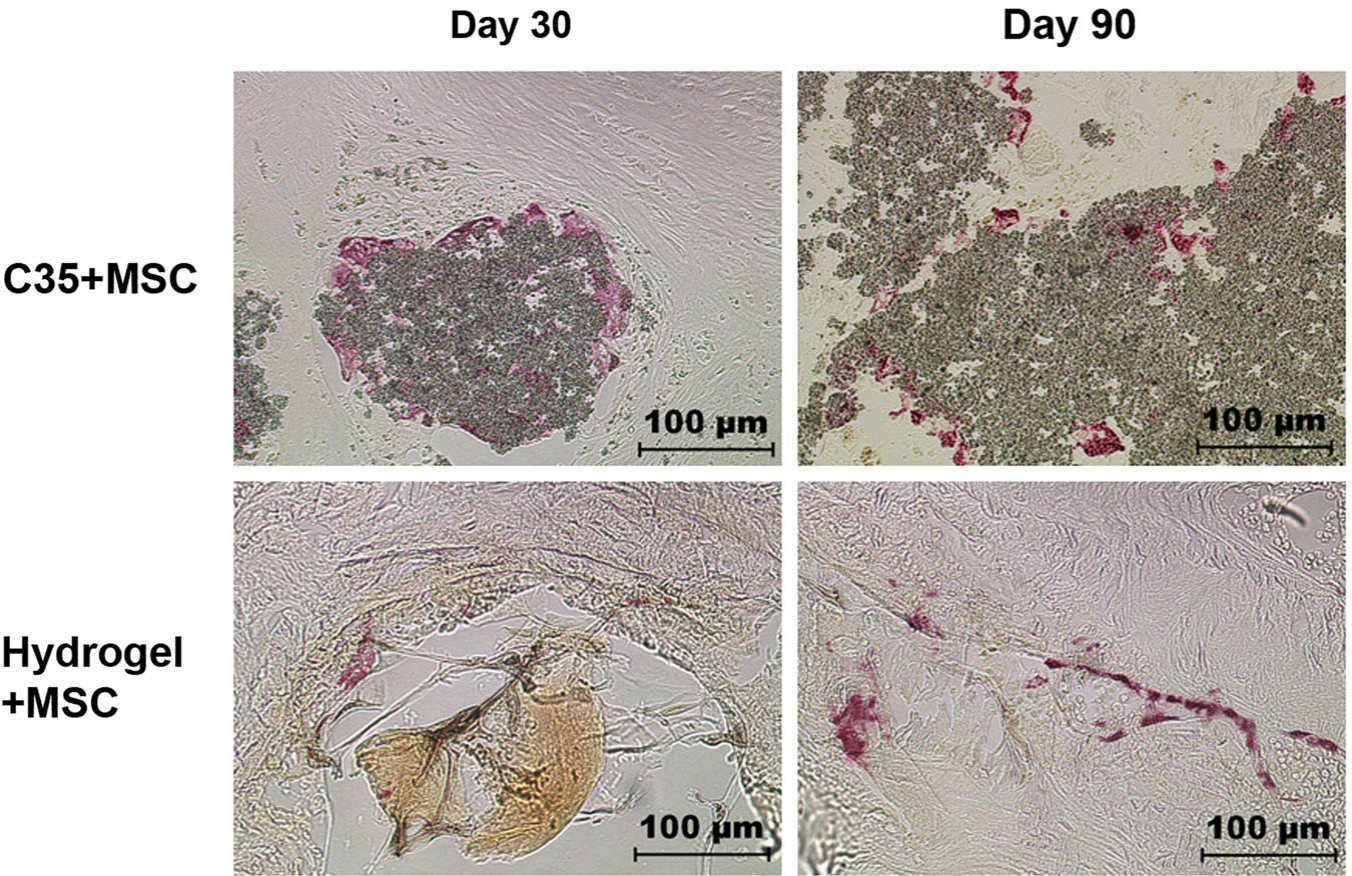
TRAP-hematoxylin staining (*Red* color: osteoclasts). Ceramic surfaces in the “C35 + MSC” group on post-surgery days 30 and 90; and newly mineralized bone in the “Hydrogel + MSC” group on days 30 and 90

**Fig. 8:**
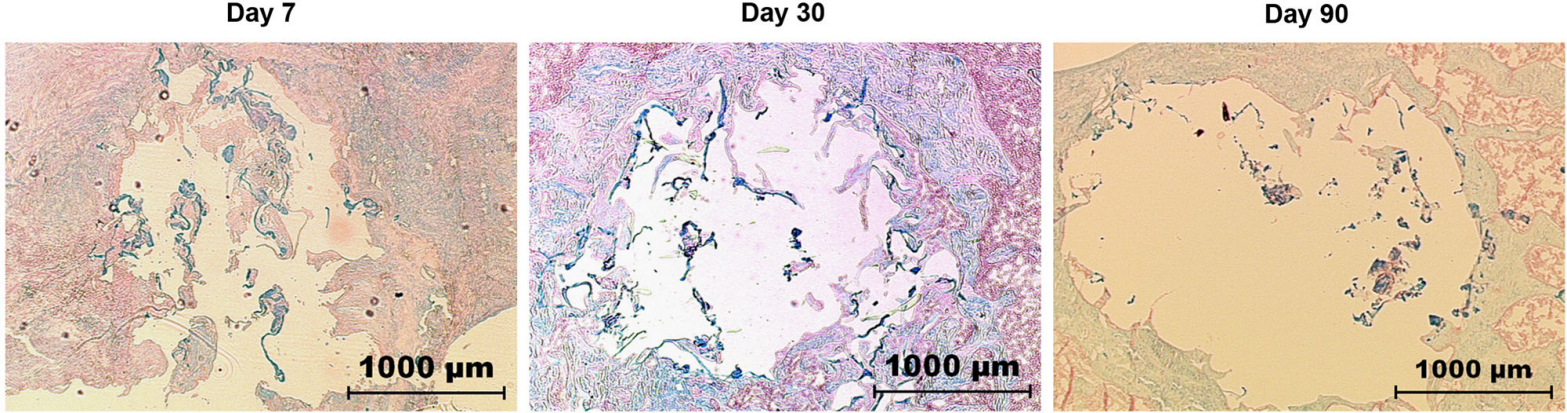
In vivo hydrogel fate overtime. Light microscopy photographs of undecalcified rat femoral defects 7, 30, or 90 days after surgery (Alcian blue staining)

Histological analysis confirmed that no resorption of C35 granules was achieved even on day 90 despite a noticeable physiological response, as suggested by the massive presence of TRAP^+^-osteoclasts at the granule surface (Fig. 7). This is in contrast with the fast hydrogel degradation that was almost completely resorbed on day 30, estimated to represent 5–10% of the initial volume (Fig. 8).

## Discussion

Increasing evidences from the literature indicate that tissue-engineering is a promising alternative to autologous bone graft for repair of critical size bone defects, but optimal scaffold remains to be defined. An ideal matrix for regenerating large bone defects should promote osteogenic differentiation of host MSCs, thanks to its own intrinsic chemical and structural properties, and promote the growth of a dense mineralized bone tissue after its implantation in the defect. Although several stem cells based products delivered through biomaterials have been tested in different models of in vivo bone repair, comparisons in a same model are rarely achieved. Here, in a rat model of large bone defect in which mechanical constraint applied to the newly formed bone is preserved, we evidenced the osteogenic properties of resorbable, soft, polysaccharide hydrogel in comparison with standard calcium-phosphate ceramic. Both hydrogel and ceramic improved bone repair by 20 and 26% of newly mineralized bone respectively, as compared to control at 3 months. The concomitant presence of ALP and TRAP-positive cells in the repair area indicates an active bone remodeling process. However, repair mechanism and resorption kinetics were strikingly different.

Using C35 ceramic, newly synthesized bone was mainly located on the granule periphery surface confirming the biocompatibility and osteoconductivity of these ceramics. Only tiny bone formation was detected in the internal pores of the C35 granules as assessed by µCT measurements, calcein labeling and histological observations. Indeed, both in vitro and in vivo bone integration into HA/TPC ceramics depend on the porosity and the pore interconnectivity of the scaffold [5, 28, 29]. According to the physical characteristics given by the manufacturer, the pore sizes of our C35 ceramics range between 100 and 400 µm and the macroporosity is about 60% (pores larger than 300 µm). These parameters should have ensured in vivo osteogenesis. Poor interconnectivity could be involved in the limited bone formation, remodeling in internal pore and subsequent observed biodegradability of HA/TCP ceramics [30]. Our histological and µCT data indicated an absence of resorption even at 3 months or in long term follow-up animals (6–7 months, data not shown) after biomaterial implantation, despite the presence of numerous TRAP positive-mature cells all around the ceramic granules (Fig. 7). This confirms several clinical investigations, in which patients treated for large varus deformity and osteoarthritis with proximal tibial opening-wedge osteotomy using porous β-TCP wedges (Ceraver) demonstrated no complete ceramic wedge resorption after a mean follow-up of 10 years [31, 32] although β-TCP ceramics have higher resorption rates than ceramics made of HA [33].

In contrast, the pullulan/dextran-based hydrogel tested herein presented impressive resorption capacity, consistent with our previous works. In a rat animal model, a porous FITC-scaffold implanted on infarcted cardiac tissue was degraded in less than a month, and only remnants of the hydrogel were seen embedded or integrated into the adjacent tissue on heart sections [34]. Physiological enzymes such as acid and alcaline phosphatases might have contributed to this in vivo degradation. Indeed, STMP cross-linking mechanism creates phosphoester linkages that are sensitive to phosphatase hydrolysis [35]. Similarly, we observed a fast degradation of porous polysaccharide hydrogel when implanted subcutaneously in adult mice [36]. This rapid hydrogel resorption was not a drawback for an efficient bone repair and supports a different repair mechanism with bone regeneration occurring on the edges of the bone defect cavity and slowly joining by the time the center of the defect up to ensure a complete bone repair in some animals. After 90 days, the newly-mineralized bone level in the medullar cavity of rats treated with hydrogel reached the same amount of newly-formed bone in the defect of animals implanted with C35 ceramics (BV/TV values ranging from 20 to 25% for either “Hydrogel” or “C35” groups on day 90). A growing interest for polymer hydrogels to enhance bone healing is arising. Soft synthetic [37] or natural polymers [38] offer several advantages including easy shaping capacity, radio-transparency and high resorption ability. The 3D structure and permeability of these polymers have a deep impact on cell physiology, modulating viability, proliferation or differentiation of various progenitor cells, as well as facilitating oxygen and nutrient delivery, or protecting soluble factors and osteoprogenitor cells [1, 24, 38–40]. We think that MSCs colonize the porous hydrogel and form aggregates of living cells in large diameter pores, that may favor interactions between cells, thereby promoting osteogenic differentiation and subsequent production of mineralized matrix [41]. Various mammalian defect models treated with polysaccharide-derived hydrogels exhibited enhanced tissue or bone repair, as reviewed in [1, 38, 42]. Recently, a novel polymer hydrogel of sugarcane molasses appeared to be a good candidate to treat calvarial bone defects in rats, in association with Bone Morphogenetic Proteins (BMPs) [43]. In patients, hyaluronan-based hydrogels associated with BMP-2 greatly enhanced the healing of critical-size cranial defects [44] or alveolar cleft defects [45], and alginate-agarose hydrogels combined with autologous chondrocytes significantly improved clinical outcome in patients suffering from chondral or osteochondral defects over a 2-year follow-up [46].

The physical and chemical properties as well as the interactions of this hydrogel with several cell lines were extensively studied [17, 18, 24, 34, 47, 48]. The hydrogel used here have also been more recently evaluated as an original base of a composite material in association with nanocrystalline hydroxyapatite particles (nHA); implanted in orthotopic preclinical models of critical size defects, in small and large animals, in three different bony sites, in goat, the hydrogel + nHA induced a highly mineralized tissue whatever the site of implantation, as well as osteoid tissue and bone tissue regeneration in direct contact to the matrix [49].

In the present study, we also assessed the influence of MSCs delivery associated with either C35 ceramics or hydrogels since MSCs are a major contributor to the natural bone repair process. Using Quantum dot^®^-labeling, we evidenced that the number of delivered MSCs engrafted in the bone defect cavity was important on day 7 but these cell numbers decreased dramatically by 30 days after implantation, independently of the considered experimental groups. Some of these MSCs appeared to be entrapped in the newly-mineralized bone and seem to locate more at the periphery of the scaffold, suggesting that (a) engrafted MSCs migrated and differentiated into mature osteoblasts to ensure bone formation and (b) a direct involvement of implanted MSCs in the bone healing process. These observations correlates with the Lalande study [48] that showed a migration of labeled adipose derived stromal cells from the center to the periphery of the hydrogel, associated with a better bone tissue regeneration process. Ninety days after implantation, labeled MSCs could not be detected anymore and the absence of MSC-enhanced bone repair at this time was consistent with the disappearance of the delivered MSCs. This observation could argue in favor of a sequential multiple MSC administration strategy all over the repair process kinetic, to support a complete bone regeneration. At day 30, MSCs delivery induced a significant increase in bone formation particularly in the hydrogel group (+61%) and furthermore, a greater osteodifferentiation capacity of cultured MSCs could be expected by expanding these progenitors in the presence of platelet lysate [20, 50, 51] rather than FGF as we performed here. We also evidenced that addition of MSCs greatly improved angiogenesis in the bone defect cavity of rats treated mainly with hydrogels, since MSCs promoted blood vessel growth within the newly-formed thick shell-like bone structure at the medullar cavity periphery. This indicated the positive influence of the rapid hydrogel resorption on bone repair. Furthermore, hydrogels could provide a protective environment for in vivo MSC survival as they could retain growth factors produced by MSCs and favor cell interactions by inducing aggregates composed of MSCs and progenitor endothelial cells. Co-cultures of hMSCs and progenitor-derived endothelial cells in the porous hydrogel induced the formation of cellular aggregates that promoted in vitro and ectopic osteogenesis in mice [47].

This is an important physiological aspect since bone is a highly metabolic tissue requiring an abundant vascular supply throughout its structure for growth, remodeling, and repair abilities [52]. Our results about the improvement of bone repair and angiogenesis obtained with the implantation of MSCs are consistent with numerous previously published studies [53].

Altogether, our results suggest that bone healing improvement provided by MSC delivery could rely on both a paracrine activity triggered by cytokines secreted from MSCs and a direct MSC involvement in bone repair process through their differentiation into mature osteoblasts responsible for bone formation. MSCs are promising candidates for successful clinical applications and several clinical trials have already been attempted as summarized by Park and colleagues [54].

## Conclusions/Perspectives

The pullulan/dextran-based hydrogel tested herein evidenced significant osteogenic properties combined with rapid resorption ability making it a promising alternative or complementary biomaterial to HA/TCP ceramics for bone repair support. Indeed, the different repair mechanisms triggered by both scaffold support the possible combination of their properties. In addition, the hydrogel capability to deliver growth factors or to entrap progenitor cells such as MSCs or endothelial progenitor cells could also be advantageously exploited and possibly enhanced by modifying hydrogel structure and stiffness. With their easy shaping capacity, hydrogel should be of particular interest for maxillo-facial and short-bone regeneration.
